# Mental rotation of hands and objects in ageing and Parkinson’s disease: differentiating motor imagery and visuospatial ability

**DOI:** 10.1007/s00221-022-06389-5

**Published:** 2022-06-09

**Authors:** Judith Bek, Stacey Humphries, Ellen Poliakoff, Nuala Brady

**Affiliations:** 1 School of Psychology, University College Dublin, Belfield, Dublin 4, Ireland; 2 Division of Neuroscience and Experimental Psychology, University of Manchester, Manchester, UK

**Keywords:** Motor imagery, Mental rotation, Hand laterality task, Parkinson’s disease, Ageing

## Abstract

Motor imagery supports motor learning and performance and has the potential to be a useful strategy for neurorehabilitation. However, motor imagery ability may be impacted by ageing and neurodegeneration, which could limit its therapeutic effectiveness. Motor imagery can be assessed implicitly using a hand laterality task (HLT), whereby laterality judgements are slower for stimuli corresponding to physically more difficult postures, as indicated by a “biomechanical constraint” effect. Performance is also found to differ between back and palm views of the hand, which may differentially recruit visual and sensorimotor processes. Older adults and individuals with Parkinson’s disease (PD) have shown altered performance on the HLT; however, the effects of both ageing and PD on laterality judgements for the different hand views (back and palm) have not been directly examined. The present study compared healthy younger, healthy older, and PD groups on the HLT, an object-based mental rotation task, and an explicit motor imagery measure. The older and PD groups were slower than the younger group on the HLT, particularly when judging laterality from the back view, and exhibited increased biomechanical constraint effects for the palm. While response times were generally similar between older and PD groups, the PD group showed reduced accuracy for the back view. Letter rotation was slower and less accurate only in the PD group, while explicit motor imagery ratings did not differ significantly between groups. These results suggest that motor imagery may be slowed but relatively preserved in both typical ageing and neurodegeneration, while a PD-specific impairment in visuospatial processing may influence task performance. The findings have implications for the use of motor imagery in rehabilitation protocols.

**Supplementary Information:**

The online version contains supplementary material available at 10.1007/s00221-022-06389-5.

## Introduction

Motor imagery (MI) is the mental simulation of movement in the absence of overt action execution (Jeannerod 1994). This simulated movement can involve both visual and kinesthetic components (Guillot et al. 2009; McAvinue and Robertson 2008) and activates a frontoparietal cortical network overlapping with areas recruited during motor execution (Hardwick et al. 2018). MI is known to facilitate movement and enhance motor learning (Malouin et al. 2013), and training with MI is widely used in sports training and rehabilitation (Schuster et al. 2011).

MI has also been utilised within rehabilitation for neurological conditions including stroke (Emerson et al. 2018; Liu et al. 2009) and Parkinson’s disease (PD) (Abbruzzese et al. 2015; Caligiore et al. 2017). However, despite promising findings from intervention studies, the ability to engage in MI and to benefit from mental practice may be affected by ageing or neurological disorders, in parallel with declining motor performance (Poliakoff 2013; Saimpont et al. 2013). Older adults have shown altered ratings of MI vividness on questionnaire-based measures (Malouin et al. 2010; Mulder 2007; although see Saimpont et al. 2015). Mental chronometry, a measure of MI based on the correspondence between physical and imagined movement times, may also be less accurate in older adults (Kuehn et al., 2018; Personnier et al. 2010), although again this is not consistently found (Scarpina et al. 2019; Wang et al. 2019). There is also evidence that mental simulation of movement may be altered in PD (Poliakoff 2013), but previous findings have differed according to the measures used. Although people with PD rate the vividness of their imagery at a similar level to age-matched controls (Heremans et al. 2011; Peterson et al. 2012), and exhibit visuomotor priming effects (Bek et al. 2018), their MI may be slowed in accordance with their physical movements (Heremans et al. 2011), and their mental chronometry may be less accurate (Scarpina et al. 2019).

**Fig. 1:**
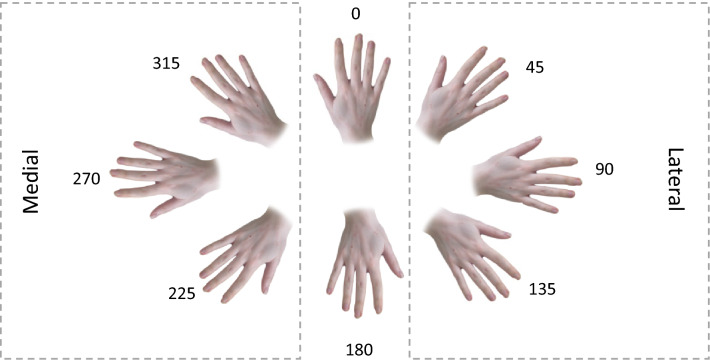
Stimuli for the hand laterality task: photographs of left and right human hands shown from both back and palm views were presented at eight different angles of rotation between 0 and 315 degrees. The example shows right-hand stimuli presented from the back view

While vividness ratings and chronometry tasks can be considered explicit measures of MI, a widely used implicit method of assessing MI ability is the hand laterality judgement task (HLT). The HLT is a mental rotation task in which participants judge the laterality of images of hands presented at increasing degrees of rotation from a canonical, viewer-centred position (Parsons 1987, 1994). Performance on this task is believed to involve covert motor simulation, and studies using neuroimaging (de Lange et al. 2006) and neurostimulation (Tomasino et al. 2005) have found evidence of motor cortex involvement during mental rotation of hands compared to objects. Several indices of behavioural performance on the HLT are also interpreted as evidence for a role of motor simulation. Parsons (1994) noted that the time taken to determine the laterality of a stimulus hand at a particular angle of rotation corresponds to the time taken to physically perform the same rotation, and that response times are longer for stimuli presented in lateral orientations (away from the midline of the body) than medial orientations (towards the midline; see Fig. 1), reflecting the biomechanical constraints affecting the corresponding physical movements. Conditions such as congenital limb absence (Funk and Brugger 2008), upper limb amputation (Nico et al. 2004), and chronic pain (Coslett et al. 2010) have been associated with slower and less accurate performance, although biomechanical constraint effects appear to be retained. Further evidence for the involvement of MI in the HLT comes from studies in which participants’ own hand or arm posture is manipulated during the task, such as by placing one hand behind the back, which is found to interfere with laterality judgements (e.g. Ionta and Blanke 2009; Ní Choisdealbha et al. 2011; Shenton et al. 2004).

Differences in HLT performance are also found according to whether stimuli depict the back or palm view of the hand. Typically, participants are slower to recognise images of the palm, and exhibit stronger biomechanical constraint effects for this view (Brady et al. 2011; de Simone et al. 2013; Nagashima et al. 2021; Ter Horst et al. 2010; Zapparoli et al. 2014). Although this may in part reflect the greater physical difficulty in rotating one’s own hands with the palm facing up (Conson et al. 2020), it has also been suggested that viewing the different surfaces of the hand may evoke different strategies, whereby kinesthetic MI is more likely to be involved in judging palm stimuli, while a more visual strategy may be engaged by the back view (Conson et al. 2020; Nagashima et al. 2021). Visual and sensorimotor familiarity may contribute to the back/palm difference, since one’s own hands are more frequently viewed from the back, while the palm may evoke stronger kinesthetic imagery due to its involvement in manual actions (Bläsing et al. 2013; Conson et al. 2020). This theory is supported by neuroimaging evidence showing greater activations in motor-related brain areas when viewing the palm, in contrast to increased activations in occipital regions for the back view (Zapparoli et al. 2014), as well as evidence of increased corticospinal excitability when judging laterality from the palm view than the back (Perruchoud et al. 2018).

It should be noted that when considering strategies in hand laterality judgement, as well as in the broader motor imagery literature (see Moran et al. 2012), there has been a lack of clarity and consistency in the terminology used to describe different forms of imagery. For example, in previous studies of the HLT, the authors have used the term “motor imagery” to imply a kinesthetic strategy, in contrast to “visual imagery” (Nagashima et al. 2021), or referred to a “motor strategy” incorporating both kinesthetic and visual MI, versus a “visual strategy” based on visual transformation (Conson et al. 2020). In the present study, “motor imagery” (MI) and “visuospatial imagery” are used, respectively, to denote (i) imagined movement that involves some degree of both kinesthetic and visual imagery, and (ii) a non-motoric form of visual imagery. We also refer to “kinesthetic MI” and “visual MI” to differentiate between components of motor imagery.

Hand laterality judgement has been found to be slowed in older adults (Devlin and Wilson, 2010; Saimpont et al. 2009; Wang et al. 2019), particularly for lateral rotations (De Simone et al. 2013; Saimpont et al. 2009) and for the non-dominant side (Saimpont et al. 2009). Older adults have also exhibited less of a clear distinction in performance between back and palm views of the hand (Nagashima et al. 2021): while younger adults consistently showed an effect of biomechanical constraints for the palm only, older adults showed more variability, with some exhibiting a medial–lateral difference for the back view. The authors interpreted this as indicating an increased tendency by older adults to use a “motor imagery” strategy for the back of the hand, as opposed to the visuospatial strategy assumed to be used by younger adults.

A small number of investigations of HLT performance in people with PD have yielded mixed results. An early study of 7 individuals with PD found an overall slowing of response times compared to age-matched controls (Dominey et al. 1995), with a particular slowing in relation to the hand most symptomatically affected. Another study comparing 12 individuals with PD, 10 healthy older adults, and 15 healthy younger adults, found slowing and reduced accuracy in the PD group (Helmich et al. 2007). Again, greater impairment was found for the most affected hand, particularly when judging hand stimuli presented at the more biomechanically difficult lateral orientations. Others have found equivalent performance between PD and healthy control groups with slightly larger sample sizes of 12 vs. 11 (van Nuenen et al. 2012) and 20 vs. 20 (Scarpina et al. 2019), although the latter study divided their PD sample into two groups of ten according to symptom lateralisation.

Altered neural activations have been found in both healthy older adults and individuals with PD during the HLT, suggesting that mechanisms underlying laterality judgement may be affected by ageing and neurodegeneration. In particular, older adults have shown increased task-related activations in occipital-temporal brain regions compared to younger adults (Zapparoli et al. 2016), and individuals with PD showed increased activity in the right extrastriate body area (EBA) and occipital-parietal areas when judging laterality corresponding to the most affected hand (Helmich et al. 2007). These findings were interpreted as suggesting increased visual processing to compensate for reduced MI ability in ageing, while individuals with PD may additionally rely on compensatory processes because of proprioceptive impairment. The involvement of the right EBA in laterality judgement by individuals with PD was further indicated by the elimination of postural congruency effects by transcranial magnetic stimulation (TMS) to this area, whereas healthy older adults  instead showed interference from stimulation to the left dorsal premotor cortex which is thought to have a key role in MI (van Nuenen et al. 2012).

The present study compared performance on the HLT in healthy younger adults, healthy older adults and individuals with PD. Given previous findings from both the HLT and explicit MI tasks, a general slowing in the older and PD groups was expected compared to the younger group, while existing evidence is more equivocal in terms of differences between healthy older and PD groups. In addition, there may be differences between groups in the use of kinesthetic or visual MI, which may be revealed in relation to different hand orientations (medial vs. lateral) or views (palm vs. back). To further understand the effects of ageing and PD on different processes in mental rotation, participants also completed a letter rotation task (Dominey et al. 1995; Scarpina et al. 2019) and an explicit measure of MI vividness (Malouin et al. 2007).

## Methods

### Participants

Participants with PD were recruited through a volunteer database, local clinics, and a PD support organisation (Parkinson’s UK). Healthy older adult (OA) participants were recruited through a volunteer database and the local community. Younger adults (YA) were recruited from the student population of the University of Manchester.

The PD group (*N* = 46) consisted of 13 females and 33 males with a mean age of 64.5 years (range 47–79, SD = 7.59). Participants had a mean disease duration of 6.7 years (range 1–20, SD = 4.2) and exhibited mild-to-moderate symptoms as indicated by scores of I–III on the Hoehn and Yahr scale and a mean score of 36.6 (range 13–55, SD = 9.86) on the motor examination of the Unified Parkinson’s Disease Rating Scale (MDS-UPDRS; Goetz et al. 2008). Symptoms were lateralised to the right side in 18 participants and the left side in 26 participants. Given previous findings relating to symptom lateralisation (Dominey et al. 1995; Helmich et al. 2007; Scarpina et al. 2019), initial analysis of data from the PD group examined the potential influence of disease severity (MDS-UPDRS motor score) and side most affected, but no significant effects on HLT or letter rotation performance were found. All participants were taking dopaminergic medication and remained on their usual medication during the study.

The OA group (*N* = 35) consisted of 21 females and 14 males with a mean age of 66 years (range 54–77, SD = 6.02), and the YA group (*N* = 30) included 22 females and 8 males with a mean age of 19.8 years (range 18–23, SD = 1.34). Participants in the OA and YA groups reported no history of neurological illness or injury.

Participants were right-handed except for three in the PD group, three in the OA group, and two in the YA group. All participants had normal or corrected-to-normal vision and hearing, and those in the OA and PD groups were screened for cognitive impairment using the Addenbrookes Cognitive Examination (ACE-III; Hsieh et al. 2013) or a brief version if they had been assessed recently for a previous study (M-ACE; Hsieh et al. 2015).^1^

The data reported here were collected as part of a larger set of studies. As such, an a priori sample size calculation was not conducted specifically for the present analysis. However, a retrospective calculation based on data from Helmich et al. (2007) provided an estimate of 11 per group to identify an overall difference in response times between groups, based on a two-tailed test at p < 0.05. While this does not account for comparisons between different orientations or interaction effects, it suggests that the present sample size is likely sufficient.

Ethical approval was obtained from a UK National Health Service Research Ethics Committee and all participants provided written informed consent.

### Stimuli

For the HLT, naturalistic stimuli were created using colour photographs of a human hand (see Fig. 1). Based on previous work (e.g. Brady et al. 2011; Dominey et al. 1995; Mibu et al. 2020), left and right hands were presented at eight different angles of rotation from an upright, canonical position (0, 45, 90, 135, 180, 225, 270 and 315 degrees), and from both back and palm views, resulting in a total of 32 different stimuli.

For the letter rotation task, stimuli were black upper case “F” or “R” characters displayed against a white background, which were presented in either canonical or mirrored form at the same eight angles of rotation as the hand.

### Procedure

The HLT was administered on a Dell 15-inch laptop with a screen refresh rate of 60 Hz using E-Prime (Psychology Software Tools, Inc., Pittsburgh, PA, United States) and response times and accuracy data were collected. A short practice block of 16 trials was first provided to ensure that participants understood the task requirements. The main task was then completed, which consisted of 6 trials of each angle (0–315) x laterality (left/right) x view (back/palm) combination (192 trials in total). The trials were split into two blocks and stimuli were fully randomised within each block. A short break was offered between the two test blocks.

To perform the task, participants were instructed to place their left and right index fingers over the G and H keys of the keyboard, respectively, and to respond as quickly and accurately as possible by pressing G when they saw a left hand and H when they saw a right hand. The stimulus image was presented against a white background and remained on screen until participants made a response, and was replaced by a central fixation cross (500 ms) between trials.

The letter rotation task was then administered using the same general procedure as the HLT, with 6 trials of each angle (0–315) x type (canonical/mirrored) x letter (F/R) combination, again resulting in a total of 192 trials. Participants were instructed to press the G key if the letter was in a canonical orientation and the H key if it was mirrored.

As a self-report measure of MI, the Kinesthetic and Visual Imagery Questionnaire (Malouin et al. 2007) was also administered. The KVIQ requires participants to physically perform, and then imagine performing, a series of basic bodily movements. Vividness of visual and kinesthetic imagery are then rated on a five-point scale to obtain subscale scores for each modality of MI.

### Data processing and analysis

#### Hand laterality task

Correct trials were analysed after the removal of excessively long (> 5000 ms) or short (< 300 ms) response times that indicated inattention or anticipatory responses. This retained an accuracy rate of 84% (compared to 89% prior to trimming of RTs), with similar overall accuracy between groups in the final dataset (PD 82.3%, OA 84.2%, YA 85%). Trials were collapsed across right and left hand stimuli, such that the angles were labelled from 0 to 315 in the clockwise direction for right hands, and in the counter-clockwise direction for left hands (Brady et al. 2011). To permit further analysis of biomechanical constraint effects, angles were recoded in terms of medial (225, 270, and 315 degrees) or lateral (45, 90, and 135 degrees) orientations (see Fig. 1).

Response times (RT) for correct trials and accuracy (% correct trials) were analysed for the trimmed dataset, with linear mixed-effects modelling (LMM) in R (R Core Team 2021) using the package lme4 (Bates et al. 2015) to examine group differences while adjusting for the influence of variation between participants. The effect of handedness was not analysed because of the very small number of left-handed participants (8 in total across groups). RT and accuracy were first analysed for the full range of angles (0–315), and then a second set of analyses examined biomechanical constraint effects in relation to medial and lateral orientations. Since previous studies have found different response profiles for back and palm views of the hand, the two views were modelled separately. In an initial set of analyses, sex was also included as predictor in the above models, but given the imbalance in sex ratios between groups, it was not possible to draw strong conclusions based on this analysis. The results are provided in supplementary materials.

#### Letter rotation task

Correct trials were trimmed for extreme RTs in the same way as above, retaining an accuracy rate of 89.5% compared to 90.6% prior to trimming (87.3% in the YA group, 96.0% in the OA group and 85.2% in the PD group). Trials were collapsed across canonical and mirrored stimuli by coding angles for canonical stimuli in the clockwise direction and angles for mirrored stimuli counter-clockwise as above. RT and accuracy were analysed using LMM.

#### KVIQ

Scores were calculated for kinesthetic and visual MI subscales of the KVIQ, which were analysed using LMM with Group and Modality (kinesthetic vs. visual) as predictors.

## Results

### Hand laterality task

**Fig. 2:**
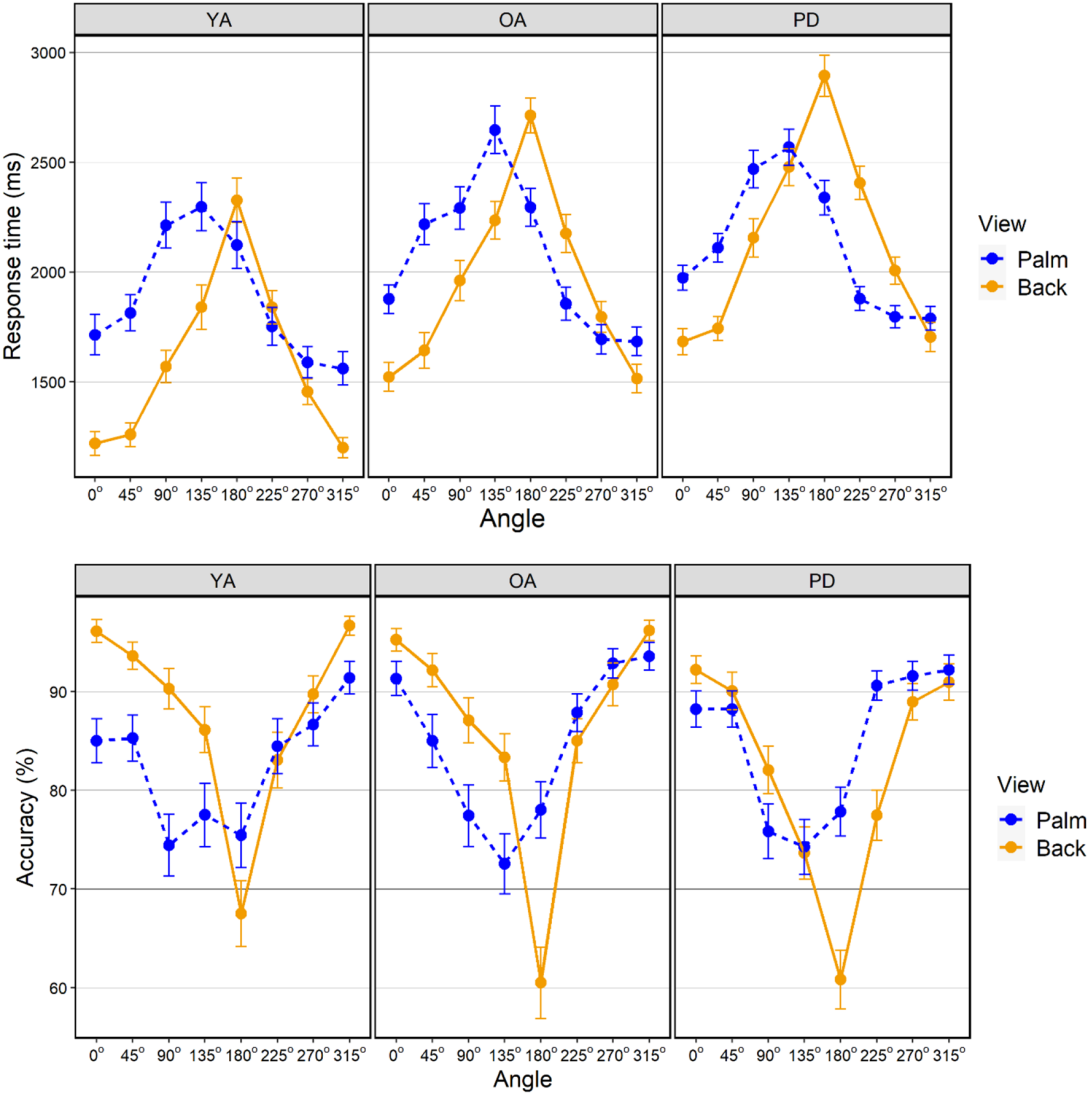
Mean RT (upper panel) and accuracy (lower panel) for each angle of rotation by group (*YA* younger adults, *OA* older adults, *PD* participants with Parkinson’s disease) and hand view (palm; back). Error bars represent SEM

RTs and accuracy across the full range of angles are illustrated in Fig. 2. For each analysis (RT and accuracy for back and palm views), a baseline model included the intercept for Participants as a random effect and Angle (0–315) as a fixed factor. The factor Group was then entered into a subsequent model. The models were compared using likelihood ratio tests to determine whether Group contributed significantly to prediction of the dependent variable. Table 1 summarises the models found to provide the best fit for RT and accuracy for back and palm views.

**Table 1:** Summary of linear mixed-effect models for RT and accuracy across the full set of angles

	Back			Palm		
Predictor	Estimate (SE)	*t*	*p*	Estimate (SE)	*t*	*p*
RT (ms)						
(Intercept)	1530.66 (81.04)	18.89	** < 0.001**	1873.36 (52.01)	36.02	** < 0.001**
Angle: 45	63.04 (30.22)	2.086	**0.037**	168.09 (32.91)	5.11	** < 0.001**
Angle: 90	408.71 (30.93)	13.22	** < 0.001**	456.63 (34.33)	13.30	** < 0.001**
Angle: 135	716.29 (31.53)	22.72	** < 0.001**	617.94 (34.32)	18.01	** < 0.001**
Angle: 180	1202.23 (34.41)	34.93	** < 0.001**	356.69 (33.95)	10.60	** < 0.001**
Angle: 225	643.64 (31.20)	20.56	** < 0.001**	− 39.05 (32.69)	− 1.19	0.23
Angle: 270	281.48 (30.40)	9.26	** < 0.001**	− 171.58 (32.44)	− 5.29	** < 0.001**
Angle: 315	− 5.79 (30.02)	− 0.19	0.85	− 188.67 (32.25)	− 5.85	** < 0.001**
Group: YA	− 359.49 (115.69)	− 3.11	**0.0024**			
Group: PD	170.24 (104.39)	1.63	0.11			
Marginal/conditional *R*^2^			0.19/0.41			0.081/0.34
Accuracy (%)						
(Intercept)	95.45 (2.07)	46.16	** < 0.001**	88.16 (1.56)	56.38	** < 0.001**
Angle: 45	− 2.55 (1.54)	− 1.66	0.098	− 2.18 (1.52)	− 1.44	0.15
Angle: 90	− 8.69 (1.55)	− 5.61	** < 0.001**	− 12.78 (1.53)	− 8.34	** < 0.001**
Angle: 135	− 14.58 (1.55)	− 9.41	** < 0.001**	− 13.87 (1.52)	− 9.10	** < 0.001**
Angle: 180	− 32.29 (1.57)	− 20.59	** < 0.001**	− 11.11 (1.52)	− 7.30	** < 0.001**
Angle: 225	− 12.95 (1.54)	− 8.38	** < 0.001**	− 0.12 (1.52)	− 0.078	0.94
Angle: 270	− 4.50 (1.54)	− 2.92	**0.0035**	2.34 (1.52)	1.54	0.12
Angle: 315	− 0.075 (1.54)	− 0.049	0.96	4.26 (1.52)	2.80	**0.005**
Group: YA	1.86 (2.65)	0.702	0.48			
Group: PD	− 4.19 (2.39)	− 1.75	0.082			
Marginal/conditional *R*^2^			0.023/0.43			0.10/0.43

The best-fitting models are presented separately for back and palm views and significant predictors are in bold

#### Response times

As illustrated in Fig. 2, RTs generally increased from 0 to 180 degrees and then decreased between 180 and 315 degrees, showing the expected effect of increasing angular displacement. However, in accordance with previous studies (Brady et al. 2011; Parsons 1994), the distribution of RTs was less symmetrical about 180 degrees for the palm than the back, reflecting a tendency for slower responses to lateral than medial angles in this view.

In modelling the back view, the effect of Angle was significant: RTs to 45, 90, 135, 180, 225, and 270 degree rotations were longer compared to the intercept of 0 degrees. Adding Group significantly increased prediction (*χ*^2^(2) = 21.504; *p* < 0.001), reflecting shorter RTs in the YA group relative to the OA group intercept, while the PD group did not significantly differ from the OA group.

For the palm view, the effect of Angle was again significant: compared to 0 degrees, RTs to 45, 90, 135, and 180 degrees were longer, while RTs to 270 and 315 degree rotations (representing medial orientations) were shorter. The model fit was not significantly increased by the addition of Group (*χ*^2^(2) = 5.01; *p* = 0.08).

#### Accuracy

Accuracy across the different rotations generally reflected the pattern for response times, with a similar asymmetrical distribution for the palm view (see Fig. 2).

For the back view, there was a significant effect of Angle in the baseline model: compared to the intercept of 0 degrees, accuracy decreased for rotations of 90, 135, 180, 225, and 270 degrees. The addition of Group significantly increased prediction (*χ*^2^(2) = 6.36; *p* = 0.042), reflecting lower accuracy in the PD group, although the main effect within the model did not reach significance at the *p* < 0.05 level (see Table 1).

For the palm view, the effect of Angle was again significant: compared to the intercept of 0 degrees, accuracy decreased for 90, 135, and 180, but increased for 315 degrees. Group did not significantly increase prediction (*χ*^2^(2) = 0.57; *p* = 0.75).

#### Biomechanical constraint effects

**Fig. 3:**
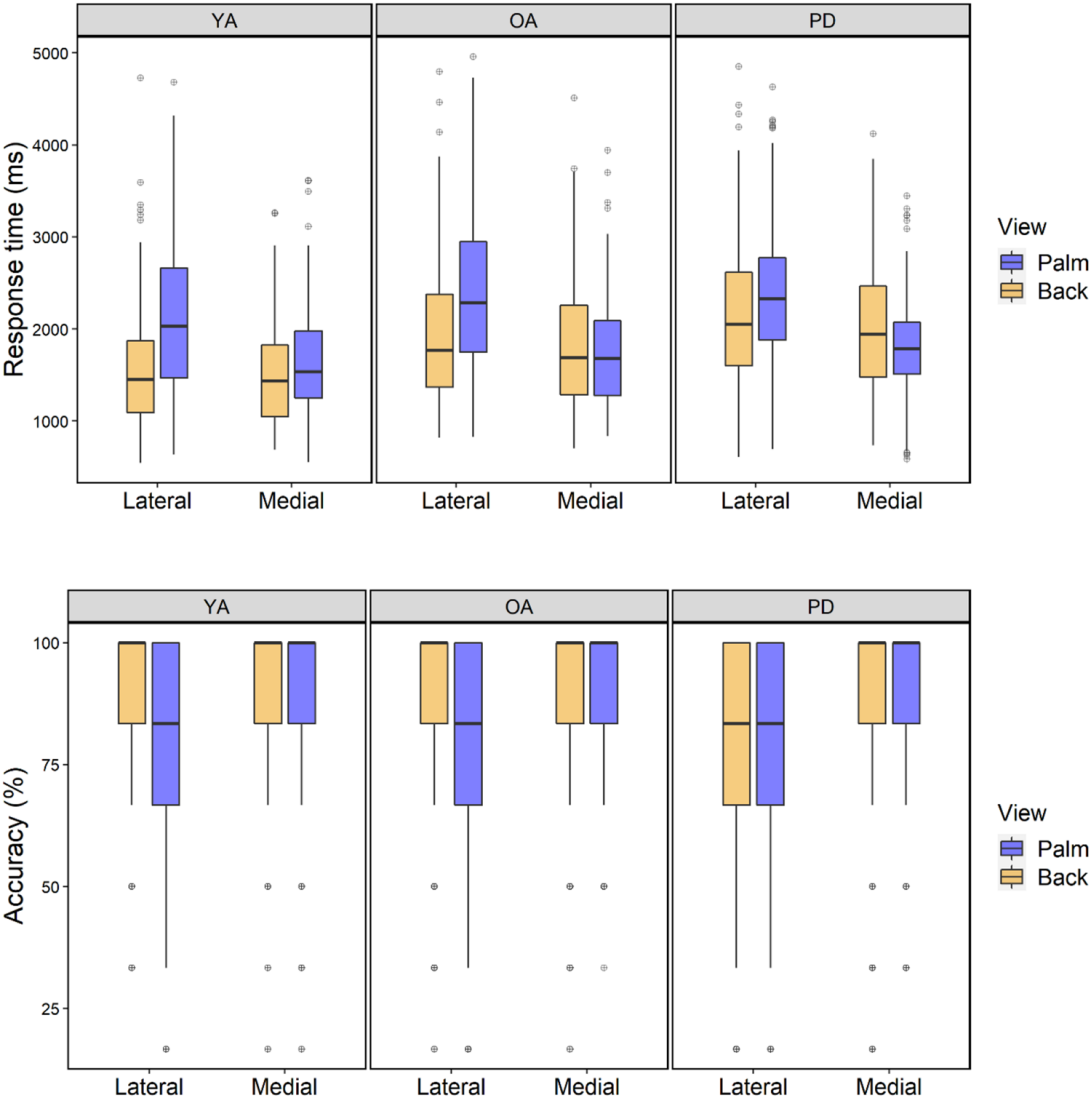
Mean RT (upper panel) and accuracy (lower panel) for lateral and medial orientations by group and view

RTs and accuracy for stimuli presented at medial (225, 270, and 315 degrees) or lateral (45, 90, and 135 degrees) orientations in each group are illustrated in Fig. 3 and the LMM analysis is summarised in Table 2.

**Table 2:** Summary of linear mixed-effect models for RT and accuracy when judging stimuli in medial and lateral orientations

Predictor	Back			Palm		
	Estimate (SE)	*t*	*p*	Estimate (SE)	*t*	*p*
RT (ms)						
(Intercept)	1922.15 (80.23)	23.96	** < 0.001**	2356.83 (85.06)	27.71	** < 0.001**
Orientation: medial	− 119.36 (34.06)	− 3.51	** < 0.001**	− 620.13 (35.33)	− 17.55	** < 0.001**
Group: PD	144.34 (106.55)	1.36	0.18	3.27 (112.85)	0.029	0.98
Group: YA	− 380.0 (117.86)	− 3.22	**0.001**	− 304.10 (125.01)	− 2.43	**0.016**
Orientation: medial *Group: PD	− 46.74 (45.63)	1.02	0.31	77.93 (46.79)	1.67	0.096
Orientation: medial *Group: YA	45.44 (49.72)	0.91	0.36	189.18 (51.87)	3.65	** < 0.001**
Marginal/conditional *R*^2^			0.054/0.28			0.087/0.33
Accuracy (%)						
(Intercept)	87.08 (1.99)	43.7	** < 0.001**	74.42 (1.80)	41.35	** < 0.001**
Orientation: medial	3.55 (1.62)	2.19	**0.028**	11.75 (0.88)	13.3	** < 0.001**
Group: PD	− 5.36 (2.64)	− 2.03	**0.044**			
Group: YA	2.92 (2.92)	1.00	0.32			
Orientation: medial *group: PD	0.52 (2.14)	0.25	0.81			
Orientation: medial *group: YA	− 3.77 (2.37)	− 1.59	0.11			
Marginal/conditional *R*^2^			0.028/0.28			0.08/0.42

The best-fitting models are presented separately for back and palm views and significant predictors are in bold

For the back view, the effect of Orientation on RT was significant, with shorter RTs to medial than lateral stimuli reflecting a biomechanical constraint effect. The addition of Group significantly increased prediction (*χ*^2^(4) = 22.64; *p* < 0.001), reflecting faster responses in the YA group consistent with the main analysis.

For the palm view, there was again a significant effect of Orientation, with shorter RTs to medially rotated stimuli. The fit of the model was significantly increased by the addition of Group (*χ*^2^(4) = 18.19; *p* = 0.001), reflecting shorter overall RTs in the YA group, as well as an Orientation*Group interaction, suggesting a smaller biomechanical constraint effect in the YA group.

A biomechanical constraint effect for the back view was further indicated by higher accuracy levels for medial than lateral rotations. Prediction was significantly increased by the addition of Group (*χ*^2^(4) = 11.25; *p* = 0.024), reflecting reduced accuracy in the PD group compared to the OA group. For the palm, accuracy was again higher for medial than lateral stimuli, but the model was not significantly improved by adding Group (*χ*^2^(4) = 5.95; *p* = 0.20).

### Letter rotation task

**Fig. 4:**
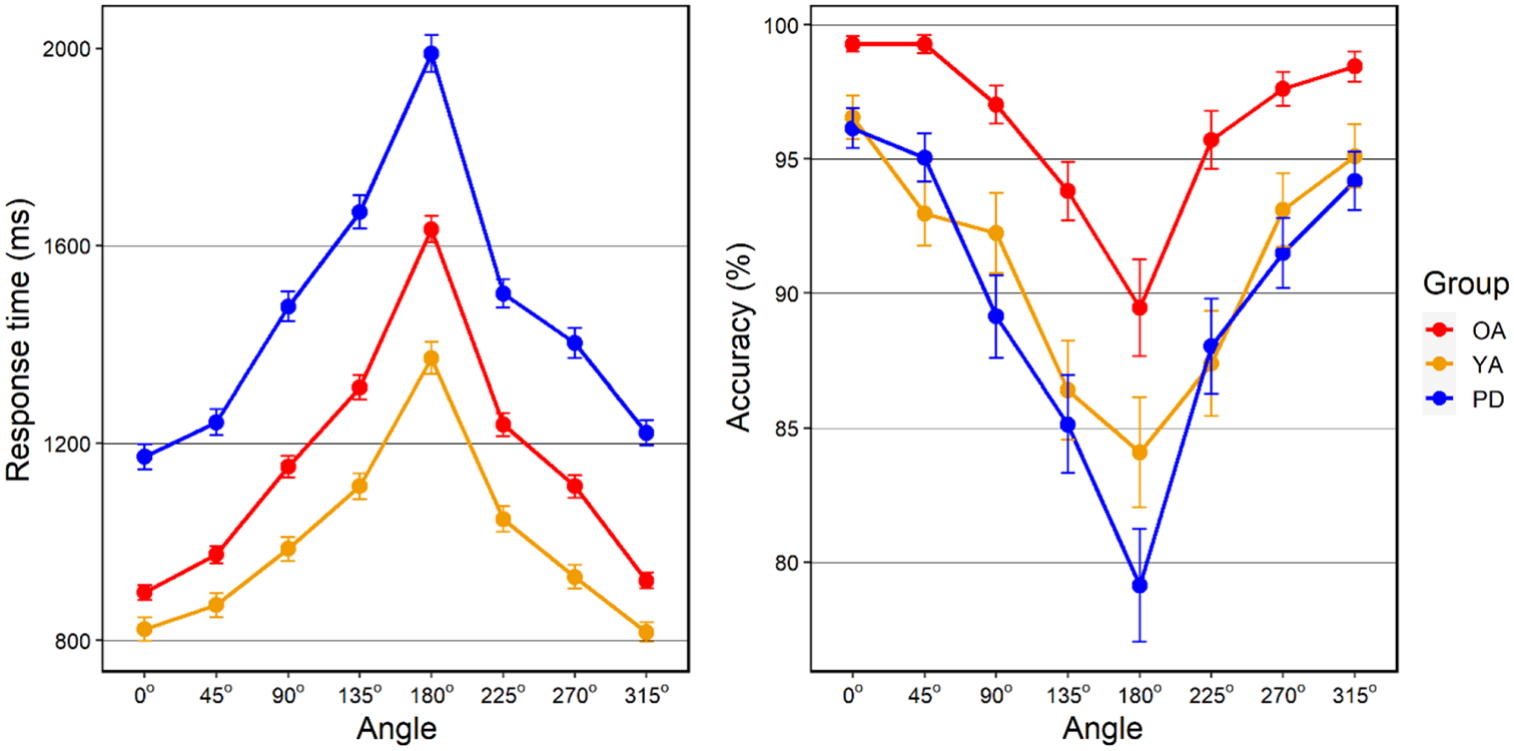
Mean RT (left panel) and accuracy (right panel) for each angle of rotation in the letter rotation task in each group (*YA* younger adults, *OA* older adults, *PD* participants with Parkinson’s disease). Error bars represent SEM

The letter rotation task was completed by a subset of 39 participants in the PD group, as well as all 35 OA participants and 30 YA participants, although data from one participant in the YA group were unusable because of a misunderstanding of the task instructions. Performance on the letter rotation task in each group is illustrated in Fig. 4 and Table 3.

**Table 3:** Summary of linear mixed-effect models for RT and accuracy in the letter rotation task

Predictor	Estimate (SE)	*t*	*p*
RT (ms)			
(Intercept)	906.26 (72.36)	12.52	** < 0.001**
Angle: 45	68.42 (17.08)	4.01	**0.037**
Angle: 90	269.42 (17.24)	15.63	** < 0.001**
Angle: 135	440.48 (17.57)	25.07	** < 0.001**
Angle: 180	750.29 (18.03)	41.61	** < 0.001**
Angle: 225	331.80 (17.44)	19.02	** < 0.001**
Angle: 270	213.39 (17.17)	12.43	** < 0.001**
Angle: 315	36.12 (17.08)	2.12	**0.034**
Group: PD	345.79 (98.50)	3.51	** < 0.001**
Group: YA	− 163.75 (106.21)	− 1.54	0.13
Marginal/conditional *R*^2^			0.15/0.44
Accuracy (%)			
(Intercept)	101.53 (1.65)	61.53 (133.28)	** < 0.001**
Angle: 45	− 1.53 (0.89)	− 1.71 (3143.49)	0.087
Angle: 90	− 4.73 (0.89)	− 5.29 (3143.49)	** < 0.001**
Angle: 135	− 9.32 (0.90)	− 10.36 (3144.33)	** < 0.001**
Angle: 180	− 13.98 (91)	− 15.37 (3145.14)	** < 0.001**
Angle: 225	− 7.23 (0.90)	− 8.06 (3143.96)	** < 0.001**
Angle: 270	− 3.39 (0.89)	− 3.80 (3143.49)	** < 0.001**
Angle: 315	− 1.53 (0.89)	− 1.71 (3143.49)	0.087
Group: PD	− 7.07 (2.12)	− 3.33 (101.50)	**0.001**
Group: YA	− 5.41 (2.29)	− 2.36 (101.33)	**0. 018**
Marginal/conditional *R*^2^			0.11/0.39

The best-fitting models are presented with significant predictors in bold

#### Response times

Similar to the hand laterality task, RTs increased as letters were rotated from 0 to 180 degrees. LMM revealed that RTs were significantly slower for all angles of rotation compared to 0 degrees. Adding Group into the model increased prediction (*χ*^2^(2) = 23.39; *p* < 0.001), reflecting longer RTs in the PD group relative to the OA group.

#### Accuracy

Compared to 0 degrees, accuracy was significantly lower for all rotations except 45 and 315 degrees. Prediction was further increased by the addition of Group (*χ*^2^(2) = 11.17; *p* = 0.0038), with both PD and YA groups being less accurate than the OA group.

#### KVIQ

KVIQ scores were significantly predicted by modality, with higher vividness ratings for visual than kinesthetic MI (*b* = 13.43, SE = 1.87, *t* (98.83) = 7.17; *p* < 0.001). Adding Group as a predictor did not significantly increase the model fit (*χ*^2^(4) = 6.15; *p* = 0.19).

## Discussion

The present study found that both healthy older adults and individuals with PD exhibited a slowing in hand laterality judgement compared to younger adults. This group difference was more pronounced when viewing the back of the hand than the palm, indicating that the results do not simply reflect general effects of ageing on response times (Verhaeghen and Salthouse 1997). Accuracy was reduced in the PD group relative to the other groups for the back view, but did not differ significantly between groups for the palm. The only previous study comparing younger, older, and PD groups found reduced speed and accuracy in the PD group but not in the healthy older controls (Helmich et al. 2007), although the sample size was smaller than in the present study, only PD participants with right-lateralised symptoms were included, and back and palm views were not analysed separately. Other studies comparing individuals with PD and age-matched controls found similar response times and accuracy between groups (Scarpina et al. 2019; van Nuenen et al. 2012).

Further analysis revealed an advantage in both response times and accuracy for hands presented in medial compared to lateral orientations, indicating the biomechanical constraint effect typically found in the HLT (Parsons 1994). When analysing this subset of angles, OA and PD groups were slower in both back and palm views and showed a larger biomechanical constraint effect than younger adults for the palm, possibly reflecting a more restricted range of physical movement. The increase in biomechanical constraint effects in both OA and PD groups is consistent with previous findings (e.g. Helmich et al. 2007; Saimpont et al. 2009; Scarpina et al. 2019), although these studies did not find differential effects for the back and palm. This similarity between groups indicates that impairments exhibited by individuals with PD in hand laterality judgement may largely reflect effects of ageing-related slowing in MI, rather than a PD-specific deficit. In contrast, performance on a test of object-based (letter) mental rotation revealed a specific impairment in the PD group, who were slower and less accurate compared to the OA group. The YA group were also less accurate than the OA group, but showed a trend towards shorter response times, suggesting a speed-accuracy trade-off (see Table 3 and Fig. 4). Together with previous findings of intact object-based rotation in older adults (e.g. Devlin and Wilson 2010), as well as the differential effects on the back and palm views in the HLT, this provides further evidence that slower performance on the HLT in older adults and PD compared to younger adults does not reflect a general slowing in mental rotation or in speed of motor response. Few studies have examined mental rotation of both hands and objects in PD. In one such study (Dominey et al. 1995), the PD group showed a greater slowing in letter rotation than hand rotation when compared with age-matched controls, consistent with the present findings. Using a similar letter-based task, Scarpina et al. (2019) found that only PD patients with right-lateralised symptoms exhibited reduced accuracy compared to age-matched controls, whereas no effects of symptom lateralisation were found in preliminary analysis of the data in the present study.

The difference in results for back and palm stimuli in the HLT echo previous findings in healthy young participants (e.g. Brady et al. 2011; Ter Horst et al. 2010), while also indicating that mechanisms involved in processing the different views may differ in susceptibility to neurodegeneration. It has been proposed that judging laterality from the back of the hand relies more on visual processes, while the palm is more likely to evoke covert motor simulation (Conson et al. 2020; Nagashima et al. 2021). Nonetheless, the present and previous findings indicate a role of MI in laterality judgements for the back of the hand as well as the palm, as reflected in biomechanical constraint effects. It is thus likely that both palm and back judgements recruit visual and kinesthetic MI, but these elements may be weighted differently for the two views (as also indicated by neuroimaging and TMS evidence; Perruchoud et al. 2018; Zapparoli et al. 2014), possibly influenced by differences in visual and sensorimotor experience of the different hand surfaces (Bläsing et al. 2013; Conson et al. 2020).

In the present study, vividness ratings (KVIQ) were higher for visual MI than kinesthetic MI, but these did not differ significantly between groups. This is consistent with previous findings comparing KVIQ scores between PD and healthy older groups (Heremans et al. 2011; Peterson et al. 2012) or healthy older and younger groups (Saimpont et al. 2015), although Malouin et al. (2010) found a reduction in “dominance” of visual over kinesthetic MI in older adults compared with younger adults. Based on principal component analysis, it has been proposed that implicit and explicit MI measures assess different dimensions of MI (Kraeutner et al. 2020). In relation to the measures used in the present study, it was suggested that the KVIQ involves the generation of MI, whereas the HLT assesses the ability to maintain and manipulate motor images. Alternatively, it is possible that effects of ageing and neurodegeneration are more likely to be found on the HLT because it is more sensitive to subtle differences in performance, or because of a slowing in the generation of MI. Functional magnetic resonance imaging (fMRI) studies have shown alterations in neural activations during the HLT (Helmich et al. 2007; Zapparoli et al. 2016) and other MI tasks (Wai et al. 2012; Wang et al. 2014) in older adults and in individuals with PD, indicating increased recruitment of cortical areas associated with visual processing. This increased activation might reflect a greater effort in generating, maintaining or manipulating visual MI, although neural activations corresponding to back and palm views in the HLT have not been compared in PD or OA groups.

If laterality judgements for palm stimuli rely more on kinesthetic MI than the back view, the smaller difference between groups in the palm view suggests that the use of kinesthetic MI may be relatively preserved in ageing and PD. Nonetheless, the increased biomechanical constraint effect for the palm in these groups might indicate that a more restricted range of motion is paralleled in MI for the corresponding movements. For the back view, despite a similar degree of slowing between OA and PD groups, older adults achieved a similar level of accuracy to younger adults, while the PD group showed a reduction in accuracy. Overall, the similarity in results between PD and OA groups suggests a general effect of ageing on the speed of mental rotation of hands, while indicating that there may be a further difficulty with visual MI in PD. Combined with the poorer performance on the letter rotation task, this may reflect a more general decline in visual processing in PD compared to typical ageing, consistent with existing evidence of visuospatial deficits in PD (e.g. Papagno and Trojano 2018; Pereira et al. 2009).

The difficulty among older adults and individuals with PD in identifying hands from the back view compared to younger adults might also relate to different visual perspectives in MI (Jackson et al. 2006). Based on studies of gesture (Humphries et al. 2016) and body representation (Conson et al. 2014), it has been proposed that people with PD have an increased tendency to represent actions from the third-person perspective (i.e. as if looking at someone else), possibly because of a difficulty in first-person MI. A reduced capacity for first-person visual MI may also occur to a lesser extent in healthy ageing (Kuehn et al. 2018; Mulder 2007; Saimpont et al. 2013). Neuroimaging and neurophysiological evidence indicates a specific involvement of the right EBA in individuals with PD during the HLT (Helmich et al. 2007; van Nuenen et al. 2012), which might reflect difficulty in adopting a first-person visual perspective (De Bellis et al. 2017; Saxe et al. 2006).

In a variation of the hand laterality task that used verbal descriptions rather than images of hand positions (Sirigu and Duhamel 2001), healthy participants showed an advantage when asked to imagine looking at their own hand as opposed to the experimenter’s hand, but this was reversed when the participant’s hands were placed in an awkward posture (behind the back). These findings indicate that visual and sensorimotor representations may be more closely connected in the first-person perspective than the third-person perspective. This suggestion is also supported by previous findings from explicit MI measures in healthy participants, which showed kinesthetic MI to be more strongly related to visual MI from the first-person (internal) than the third-person (external) perspective, particularly when the third-person represented “other” rather than “self” (Callow and Hardy 2007; Miller and Saygin 2013). By extension, for more familiar hand views or postures in the HLT (e.g. the back view), a combination of visual and kinesthetic MI may facilitate recognition, whereas a greater reliance on kinesthetic MI for the less visually familiar palm view results in slower overall responses and increased biomechanical constraint effects. A greater impairment of visuospatial processing in individuals with PD could thus impact on the advantage typically gained by integrating visual and kinesthetic representations in MI.

The nature of hand laterality judgement in ageing and neurodegenerative conditions could be further investigated through behavioural manipulations such as instructing participants to focus on visual versus kinesthetic MI, or to adopt a first-person versus third-person strategy (as in Sirigu and Duhamel 2001). Further neurophysiological studies could also be useful to identify neural processes underlying HLT performance using TMS (Kraeutner et al. 2019) or transcranial direct current stimulation (Veldema et al. 2021), particularly comparing back and palm views.

The present findings have relevance for the use of MI as a technique to improve movement. In healthy participants, training in hand laterality judgement has been found to enhance performance, with corresponding neuroplastic effects (Berneiser et al. 2018), indicating that mental hand rotation practice may alter motor representations. More broadly, the findings support the use of kinesthetic MI in rehabilitation and therapeutic activities, particularly when combined with action observation, which may circumvent difficulties with visual MI (e.g. Bek et al. 2021; for review see Eaves et al. 2016). Training in visual MI and perspective-taking may also enhance the effectiveness of MI interventions for individuals with PD.

An important limitation of this and previous studies of MI ability in PD is that the sex ratio between groups was not matched and the proportion of females in the PD group was relatively small (28%), reflecting the greater incidence of PD among males in the general population (Wooten et al. 2004). In young healthy participants, evidence suggests a male advantage in hand laterality judgement for the palm view while females show an advantage for the back of the hand (Conson et al. 2020), but sex differences in the HLT have not previously been reported in older adults or individuals with PD. Initial analysis of data from the present study indicated potential sex differences in the PD group, both in hand laterality judgement for the back view and in letter rotation (see supplementary material), but these findings need to be investigated further in a larger sample. Identifying sex differences in MI ability in PD has important implications for rehabilitation techniques based on motor simulation, particularly in light of evidence that males and females may be differently affected by PD (Miller and Cronin-Golomb 2010; Reekes et al. 2020; Subramanian et al. 2022).

In summary, the ability to perform mental hand rotation appears to be influenced by a general ageing effect, possibly reflecting greater physical limitations on movement. In mild-to-moderate PD, although performance on MI tasks appears to be generally similar to that of healthy older adults, there may be particular difficulties in drawing upon first-person visual representations of the body or integrating visual and kinesthetic elements of motor imagery.

## Supplementary Information

Below is the link to the electronic supplementary material.

Supplementary file1 (PDF 616 KB)

## Data Availability

The datasets analysed for the current study are available from the corresponding author on reasonable request.

## Footnotes

1 While three participants in the PD group scored close to the cutoff, excluding their data did not alter any of the results, so these individuals were retained in the final analysis.
