# Supplementary material for: Mental rotation of hands and objects in ageing and Parkinson’s disease: differentiating motor imagery and visuospatial ability
Source: Exp Brain Res. 2022 Jun 9;240(7-8):1991–2004. doi: 10.1007/s00221-022-06389-5 (PMC9288383; doi:10.1007/s00221-022-06389-5)
Supplement: Supplementary file 1 — Supplementary file1 (PDF 616 KB) [file 221_2022_6389_MOESM1_ESM.pdf]

Analysis of sex differences in hand and letter rotation tasks

RTs and accuracy across the full range of angles are illustrated in Figure S1. For each analysis (RT and accuracy for back and palm views), a baseline model included the intercept for Participants as a random effect and Angle (0-315) as a fixed factor. Group and Sex and the interaction between these factors were then entered into subsequent models. Each model was compared to the previous less complex one using likelihood ratio tests to determine whether it contributed significantly to prediction of the dependent variable. Table S1 summarises the models found to provide the best fit for RT and accuracy.

RT across angles

As illustrated in Figure 2, RTs generally increased from 0 to 180 degrees and then decreased between 180 and 315 degrees, showing the expected effect of increasing angular displacement. However, in accordance with previous studies (Brady et al., 2011; Parsons, 1994), the distribution of RTs was less symmetrical about 180 degrees for the palm than the back, reflecting a tendency for slower responses to lateral than medial angles in this view.

In modelling the back view, the effect of Angle was significant: RTs to 45, 90, 135, 180, 225 and 270 degree rotations were longer compared to the intercept of 0 degrees. Adding Group significantly increased prediction ($\chi^2(2) = 21.504$; $p<.001$), reflecting shorter RTs in the YA group relative to the OA group intercept, while the PD group did not significantly differ from the OA group. The addition of Sex did not further increase the fit of the model ($\chi^2(3) = 2.19$; $p=.53$).

For the palm view, the effect of Angle was again significant: compared to 0 degrees, RTs to 45, 90, 135, and 180 degrees were longer, while RTs to 270 and 315 degree rotations (representing medial orientations) were significantly shorter. The model fit was not significantly increased by the addition of Group ($\chi^2(2) = 5.01$; $p=.08$) or Sex ($\chi^2(2) = 5.81$; $p=.054$).

Accuracy across angles

Accuracy across different rotations generally reflected the pattern for response times, with a similar asymmetrical distribution for the palm view (see Figure S1).

For the back view, there was a significant effect of Angle in the baseline model: compared to the intercept of 0 degrees, accuracy decreased for rotations of 90, 135, 180, 225 and 270 degrees. The addition of Group significantly increased prediction ($\chi^2(2) = 6.36$; $p = .042$), reflecting lower accuracy in the PD group, although the main effect within the model did not reach significance at the $p<.05$ level. Sex did not further increase the fit of the model ($\chi^2(3) = .44$; $p = .93$).

For the palm view, the effect of Angle was again significant: compared to the intercept of 0 degrees, accuracy decreased for 90, 135 and 180, but increased for 315 degrees. Group did not significantly increase prediction ($\chi^2(2) = .57$; $p = .75$), but a significant effect of Sex ($\chi^2(1) = 7.56$; $p = .006$), indicated higher accuracy in males.

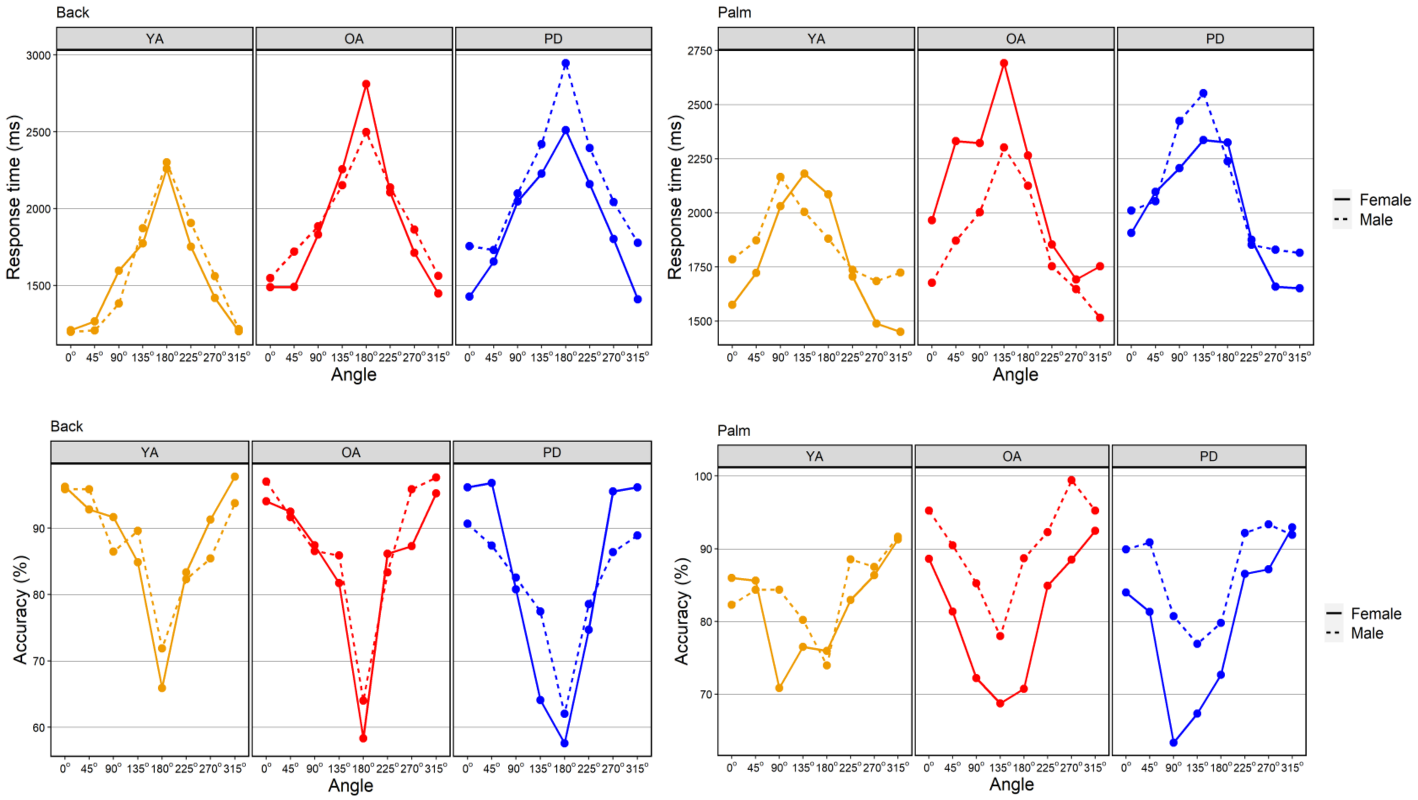

Figure S1. Mean RT (upper panel) and accuracy (lower panel) for each angle of rotation by group (younger adults, YA; older adults, OA; participants with Parkinson's disease, PD) and sex for the back view (left) and palm view (right).

Table S1. Summary of linear mixed-effect models for RT and accuracy across the full set of angles. The best-fitting models are presented separately for back and palm views and significant predictors are highlighted in bold.

	Back			Palm		
Predictor	*Estimate (SE)*	*t*	*p*	*Estimate (SE)*	*t*	*p*
**RT (ms)**						
(Intercept)	1530.66 (81.04)	18.89	**<.001**	1873.36 (52.01)	36.02	**<.001**
Angle: 45	63.04 (30.22)	2.086	**.037**	168.09 (32.91)	5.11	**<.001**
Angle: 90	408.71 (30.93)	13.22	**<.001**	456.63 (34.33)	13.30	**<.001**
Angle: 135	716.29 (31.53)	22.72	**<.001**	617.94 (34.32)	18.01	**<.001**
Angle: 180	1202.23 (34.41)	34.93	**<.001**	356.69 (33.95)	10.60	**<.001**
Angle: 225	643.64 (31.20)	20.56	**<.001**	-39.05 (32.69)	-1.19	.23
Angle: 270	281.48 (30.40)	9.26	**<.001**	-171.58 (32.44)	-5.29	**<.001**
Angle: 315	-5.79 (30.02)	-.19	.85	-188.67 (32.25)	-5.85	**<.001**
Group: YA	-359.49 (115.69)	-3.11	**.0024**			
Group: PD	170.24 (104.39)	1.63	.11			
Marginal/Conditional R^2			.19 / .41			.081 / .34
**Accuracy (%)**						
(Intercept)	95.45 (2.07)	46.16	**<.001**	88.16 (1.56)	56.38	**<.001**
Angle: 45	-2.55 (1.54)	-1.66	.098	-2.18 (1.52)	-1.44	.15
Angle: 90	-8.69 (1.55)	-5.61	**<.001**	-12.78 (1.53)	-8.34	**<.001**
Angle: 135	-14.58 (1.55)	-9.41	**<.001**	-13.87 (1.52)	-9.10	**<.001**
Angle: 180	-32.29 (1.57)	-20.59	**<.001**	-11.11 (1.52)	-7.30	**<.001**
Angle: 225	-12.95 (1.54)	-8.38	**<.001**	-.12 (1.52)	-.078	.94
Angle: 270	-4.50 (1.54)	-2.92	**.0035**	2.34 (1.52)	1.54	.12
Angle: 315	-.075 (1.54)	-.049	.96	4.26 (1.52)	2.80	**.005**
Group: YA	1.86 (2.65)	.702	.48			
Group: PD	-4.19 (2.39)	-1.75	. 082			
Sex: Male				6.48	2.80	**.006**
Marginal/Conditional R^2			.023 / .43			.13 / .43

Note: Estimates and associated statistics for factors are only reported where their inclusion contributes significantly to the model.

Biomechanical constraint effects

RTs and accuracy for medial and lateral orientations in each group are illustrated in Figure S2 and the LMM analysis is summarised in Table S2.

For the back view the effect of Orientation on RT was significant, with shorter RTs to medial than lateral stimuli reflecting a biomechanical constraint effect. The addition of Group increased prediction ($\chi^2(4) = 22.64$; p<.001), reflecting faster responses in the YA group consistent with the main analysis. Sex also increased prediction ($\chi^2(6) = 16.65$; p=.011): a three-way interaction between Orientation, Group and Sex indicated a reduced biomechanical constraint effect in the PD group among males. For the palm view, there was again a significant effect of Orientation, with shorter RTs to medial rotations. The fit of the model was increased by the inclusion of Group ($\chi^2(4) = 18.19$; p=.001), reflecting shorter overall RTs in the YA group, as well as an Orientation*Group interaction, suggesting a smaller biomechanical constraint effect in the YA group. The addition of Sex further increased prediction ($\chi^2(6) = 14.78$; p=.022): males showed shorter RTs overall, while an interaction of Orientation*Sex indicated a smaller biomechanical constraint effect in males than females.

For stimuli viewed from the back, a biomechanical constraint effect was further indicated by increased accuracy for medial rotations. Prediction was increased by the addition of Group ($\chi^2(4) = 11.25$; p = .024), reflecting reduced accuracy in the PD group, but there was no significant effect of adding Sex into the model ($\chi^2(6) = 5.77$; p = .45). For the palm, accuracy was again higher for stimuli in medial rotations. The model was not significantly improved by adding Group ($\chi^2(4) = 5.95$; p = .20), but was increased by the addition of Sex ($\chi^2(2) = 11.58$; p = .003), with males showing greater overall accuracy but a reduced advantage for medial rotations, suggesting a smaller effect of biomechanical constraints.

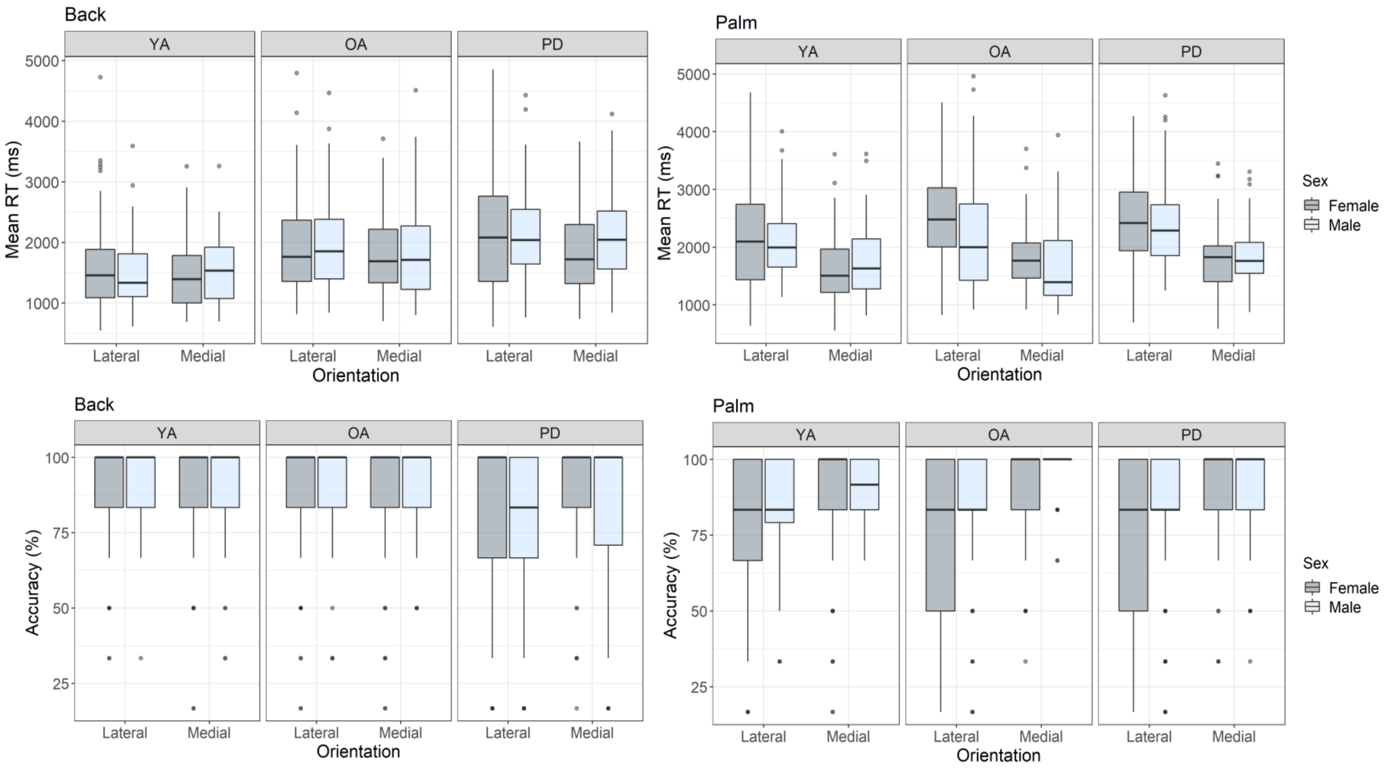

Figure S2. Mean RT (upper panel) and accuracy (lower panel) for lateral and medial orientations by group and sex for the back view (left) and palm view (right).

Table S2. Summary of linear mixed-effect models for RT and accuracy in judging stimuli in medial and lateral orientations. The best-fitting models are presented separately for back and palm views and significant predictors are highlighted in bold.

	Back			Palm		
Predictor	*Estimate (SE)*	*t*	*p*	*Estimate (SE)*	*t*	*p*
**RT (ms)**						
(Intercept)	1875.03 (102.65)	18.27	**<.001**	2502.73 (109.14)	22.93	**<.001**
Orientation: Medial	-110.81 (43.95)	-2.52	**.012**	-716.8 (46.76)	-15.33	**<.001**
Group: PD	128.98 (166.26)	.78	.44	-156.03 (176.83)	-.88	.38
Group: YA	-317.012 (143.28)	-2.21	**.029**	-456.47 (152.18)	-3.0	**.003**
Sex: Male	117.93 (162.44)	.73	.47	-351.42 (171.70)	-2.05	**.043**
Orientation: Medial * Group:PD	-115.92 (71.92)	-1.61	.11	124.93 (76.43)	1.64	.102
Orientation: Medial * Group:YA	-7.01 (60.93)	-.12	.91	254.22 (64.62)	3.93	**<.001**
Orientation: Medial * Sex:Male	-21.46 (252.66)	-.31	.76	224.33 (71.29)	3.15	**.0012**
Group: PD * Sex: Male	-30.13 (224.12)	-.13	.89	371.96 (237.32)	1.57	.12
Group: YA * Sex: Male	-176.86 (252.66)	-.70	.49	378.02 (267.26)	1.41	.16
Orientation: Medial * Group:PD * Sex: Male	236.87 (96.67)	2.45	**.014**	-157.28 (100.0)	-1.57	.12
Orientation: Medial * Group:YA * Sex: Male	187.48 (107.47)	1.74	.081	-109.81 (110.78)	-.99	.32
Marginal/ Conditional R^2			.06 / .29			.093 / .33

Table S2 (continued).

	Back			Palm		
Predictor	*Estimate (SE)*	*t*	*p*	*Estimate (SE)*	*t*	*p*
**Accuracy (%)**						
(Intercept)	87.08 (1.99)	43.7	**<.001**	74.42 (1.80)	41.35	**<.001**
Orientation: Medial	3.55 (1.62)	2.19	**.028**	13.58 (1.24)	10.93	**<.001**
Group: PD	-5.36 (2.64)	-2.03	**.044**			
Group: YA	2.92 (2.92)	1.00	.32			
Orientation: Medial * Group:PD	.52 (2.14)	.25	.81			
Orientation: Medial * Group:YA	-3.77 (2.37)	-1.59	.11			
Sex: Male				8.40 (2.56)	3.29	**.0013**
Sex: Male * Orientation: Medial				-3.70 (1.76)	-2.10	**.036**
Marginal/ Conditional R^2			.028 / .28			.11 / .42

Note: Estimates and associated statistics for factors are only reported where their inclusion contributes significantly to the model.

Letter rotation task

The letter rotation task was completed by a subset of 39 participants in the PD group, as well as all 35 OA participants and 30 YA participants, although data from one participant in the YA group was unusable because of a misunderstanding of the task instructions. Performance on the letter rotation task in each group is illustrated in Figure S3 and Table S3.

Similar to the hand task, RTs increased as letters were rotated from 0 to 180 degrees. LMM revealed that RTs were significantly slower for all angles of rotation compared to 0 degrees. Adding Group into the model increased prediction ($\chi^2(2) = 23.39$; $p < .001$), reflecting longer RTs in the PD group, but not the YA group, relative to the OA group. Sex also contributed to the model ($\chi^2(3) = 11.53$; $p = .0092$), reflecting an interaction between Group and Sex: as evident in Figure 4, this final model revealed that males in the PD group were slower, while females showed similar performance to those in the OA group.

Compared to 0 degrees, accuracy was significantly lower for all rotations except 45 and 315 degrees. Prediction was further increased by the addition of Group ($\chi^2(2) = 11.17$; $p = .0038$), with both PD and YA groups being less accurate than the OA group, while Sex did not further affect the fit of the model ($\chi^2(1) = .82$; $p = .36$).

Table S3. Summary of linear mixed-effect models for RT and accuracy in the letter rotation task. The best-fitting models are presented with significant predictors highlighted in bold.

Predictor	*Estimate (SE)*	*t*	*p*
**RT (ms)**			
(Intercept)	932.03 (87.98)	10.59	**<.001**
Angle: 45	68.42 (17.08)	4.01	**.037**
Angle: 90	269.41 (17.24)	15.63	**<.001**
Angle: 135	440.5 (17.57)	25.07	**<.001**
Angle: 180	750.37 (18.03)	41.61	**<.001**
Angle: 225	331.87 (17.44)	19.03	**<.001**
Angle: 270	213.41 (17.17)	12.43	**<.001**
Angle: 315	36.12 (17.08)	2.12	**.034**
Group: PD	-4.26 (144.71)	-.03	.997
Group: YA	-157.62 (122.02)	-1.29	.196
Sex: Male	-64.52 (137.96)	-.47	.64
Group: PD * Sex: Male	533.01 (195.70)	2.72	**.006**
Group: YA * Sex: Male	-67.75 (221.72)	-.31	.76
Marginal/ Conditional R^2			.18 / .44
**Accuracy (%)**			
(Intercept)	101.53 (1.65)	61.53 (133.28)	**<.001**
Angle: 45	-1.53 (.89)	-1.71 (3143.49)	.087
Angle: 90	-4.73 (.89)	-5.29 (3143.49)	**<.001**
Angle: 135	-9.32 (.90)	-10.36	**<.001**
Angle: 180	-13.98 (91)	-15.37	**<.001**
Angle: 225	-7.23 (.90)	-8.06 (3143.96)	**<.001**
Angle: 270	-3.39 (.89)	-3.80 (3143.49)	**<.001**
Angle: 315	-1.53 (.89)	-.1.71 (3143.49)	.087
Group: PD	-7.07 (2.12)	-3.33 (101.50)	**.001**
Group: YA	-5.41 (2.29)	-2.36 (101.33)	**. 018**
Marginal/ Conditional R^2			.11 / .39

Note: Estimates and associated statistics for factors are only reported where their inclusion contributes significantly to the model.

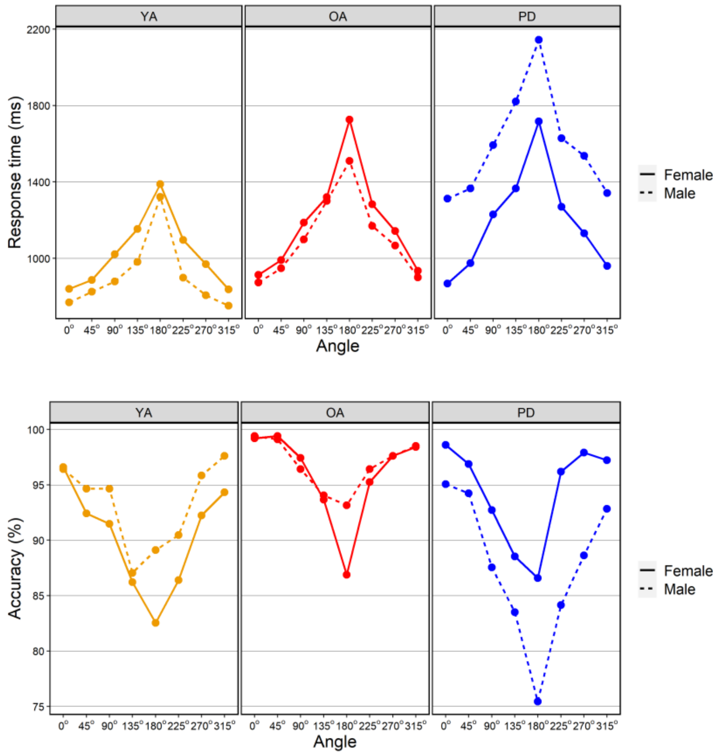

Figure S3. Mean RT (upper panel) and accuracy (lower panel) for each angle of rotation in the letter rotation task by group (younger adults, YA; older adults, OA; participants with Parkinson's disease, PD) and sex.
